# Orthogonal Chirp-Based Ultrasonic Positioning

**DOI:** 10.3390/s17050976

**Published:** 2017-04-27

**Authors:** Mohammad Omar Khyam, Shuzhi Sam Ge, Xinde Li, Mark Pickering

**Affiliations:** 1 Department of Electrical and Computer Engineering, National University of Singapore, Singapore 117580, Singapore; samge@nus.edu.sg (S.S.G.); xindeli@seu.edu.cn (L.X.); 2 Key Laboratory of Measurement and Control of CSE, School of Automation, Southeast University, Ministry of Education, Nanjing 210096, China; 3 School of Engineering and Information Technology, University of New South Wales, Canberra, Campbell ACT 2612, Australia; m.pickering@unsw.edu.au

**Keywords:** multiple access, time-of-flight, orthogonal chirp, ultrasonic indoor positioning

## Abstract

This paper presents a chirp based ultrasonic positioning system (UPS) using orthogonal chirp waveforms. In the proposed method, multiple transmitters can simultaneously transmit chirp signals, as a result, it can efficiently utilize the entire available frequency spectrum. The fundamental idea behind the proposed multiple access scheme is to utilize the oversampling methodology of orthogonal frequency-division multiplexing (OFDM) modulation and orthogonality of the discrete frequency components of a chirp waveform. In addition, the proposed orthogonal chirp waveforms also have all the advantages of a classical chirp waveform. Firstly, the performance of the waveforms is investigated through correlation analysis and then, in an indoor environment, evaluated through simulations and experiments for ultrasonic (US) positioning. For an operational range of approximately 1000 mm, the positioning root-mean-square-errors (RMSEs) &90% error were 4.54 mm and 6.68 mm respectively.

## 1. Introduction

The location of a radiating ultrasonic source can be determined in three-dimensional (3D) space using information regarding its distances from at least three reference points, the locations of which are known, providing that the configuration of these reference points is adequate. This technique has been extensively used in research and production fields in many and varied applications, such as indoor positioning [1,2,3,4,5,6,7], robot navigation [8,9] and human pose estimation [10].

Generally, in an UPS the distance information is obtained using the travel time information of the physical signal propagating between a transmitter and receiver, known as the time-of-flight (TOF). The most widely used TOF estimation methods are phase detection, threshold detection and cross-correlation. In phase detection, the phase difference between transmitted and received signal is calculated to estimate the TOF. The main problem with this approach is the existence of ambiguity when the measured range is larger than the wavelength (λ) of the transmitted signal. Hence, without ambiguity, it can measure the maximum phase difference of 2π radians, in other words, a distance of up to a wavelength (λ) [11]. In threshold detection approach the TOF is measured by triggering the event when the received signal exceeds a predefined threshold level for the first time which, of course, must be above the noise level. Although this is computationally simple and can be implemented with low-cost single-frequency US transducers, for low signal-to-noise ratio (SNR) signals, it is not the most suitable method. This is because, on average, it estimates a false positive TOF compared with the actual one [12], in other word, it is more probable to estimate a false positive location.

A more standard and proper TOF estimation technique is cross-correlation, in which transmitted and received signals are cross-correlated to produce the maximum value at the time delay, that performs better than the threshold technique for low SNR signals. It is considered the optimal TOF estimation technique as it uses all the information contained in the signals [12]. However, it performs poorly for estimating the TOF of a single-tone signal because as, in a particular signal length, there are several cycles which produce very similar peaks adjacent to the main one when cross-correlated with the received signal, false peaks may be detected in a noisy environment [11]. Cross-correlation provides improved accuracy when the waveform is not a single-tone signal but a frequency-modulated (FM) one, such as a linear chirp, because cross-correlation produces a narrower cross-correlated peak at the time delay for a chirp signal [11].

Therefore, as a chirp-based cross-correlation technique provides more accurate distance estimations through TOF measurements, it has been extensively used in UPSs. However, when a chirp signal is used for positioning, existing UPSs suffer from problems due to signal interference [1,5]. For example, if the useful frequency range of an UPS is 35 kHz to 45 kHz and multiple transmitters transmit the same band of signals simultaneously, they will interfere with each other at the receiving end. Therefore, to support multiple access in a chirp-based UPS, the transmitted chirp signals must be orthogonal. This can be achieved using either time-division multiplexing (TDM) or frequency-division multiplexing (FDM). In the TDM technique, orthogonality is maintained by transmitting the same pulse from collocated transmitters at different times, i.e., one after another, with proper intervals between them to avoid signal interference at the receiving end. However, as this leads to a slower update rate because only one transmitter is allowed to send at a time, the number of location updates possible in a given time interval is limited. Therefore, this is not an efficient solution for applications for which simultaneous transmission is a prerequisite. In the FDM technique, orthogonality is maintained by ensuring that separate sources are spaced sufficiently far apart in the frequency domain so that no interference occurs, i.e., the bandwidth to the transmitters is split. However, this deteriorates the cross-correlation performance, the accuracy of which depends on the bandwidth of the chirp [11].

Some methods such as direct sequence spread spectrum (DSSS) [4], code division multiple access (CDMA) [3] and frequency hopped spread spectrum (FHSS) [2] have been proposed using broadband transducers that are more expensive than narrowband transducers. A summary of the broadband UPSs can be found in [6,7]. Please note that as in this paper we proposed multiple access chirp-based ultrasonic positioning, here we only described similar systems and to the best of our knowledge, only one previous paper [13] has demonstrated the use of pseudo-orthogonal chirp waveforms for UPS. This system adapted the use of chirp rates as a mechanism for assigning uniquely modulated chirp signals to transmitters from wireless data communications [14] to UPS applications. As it uses the diversity of the chirp rates for multiple access in an UPS, all the advantages of the classical chirp waveform are presented into the system. However, the problem of this approach is that due to the symmetricity of the chirp rate, when the number of transmitters in the system is increased the multiple-access interference (MAI) is also increased.

In this paper, to facilitate multiple access transmission in a chirp-based UPS, we present orthogonal chirp waveforms, in which multiple transmitters can simultaneously transmit chirp signals, therefore, it can utilize the entire available frequency spectrum efficiently. Moreover, the proposed orthogonal chirp waveforms have all the advantages of classical chirp waveform. The fundamental idea behind this approach is to utilize the oversampling methodology of OFDM modulation and orthogonality of the discrete frequency components of a chirp waveform.

The rest of this paper is organized as follows: Section 2 provides a general description of UPS; Section 3 provides a mathematical model which shows how an UPS suffers from the multiple-access problem; Section 4 describes details of our proposed ultrasonic multiple access system; Section 5 presents an assessment of the system’s performance; Section 6 gives simulation results for a passive mobile architecture; Section 7 illustrates experimental determination of the precision of the proposed system; Section 8 provides experimental results; and Section 9 offers some conclusions drawn from this study.

## 2. Ultrasonic Positioning

The process of determining the current location of a target(s) within given coordinates using a location system is called localization which consists of two phases [15]: (i) measurement phase (determines the distances between the reference points and target(s)) and; (ii) positioning phase (exploits the measured information to calculate the position of the target(s)). Mostly, existing UPSs use a cross-correlation technique in the measurement phase and a lateration algorithm in the positioning phase, as discussed in the following subsections.

### 2.1. Lateration

The position of a target in 3D space can be determined by measuring its distance from at least three reference points the locations of which are known, providing that the reference points are placed in a single plane non-collinearly (i.e., reference points that do not all lie on a single straight line) [16]. The distance from the target to each reference point is taken as the radius of a sphere centered at that reference point. These spheres will intersect at just two points. One of these intersecting points can usually be discarded as it is located in a physically impossible position (e.g., below the ground or outside the room) so the remaining intersecting point is taken as the position of the target.

If we denote the coordinates of the unknown location of the target as (x,y,z), the coordinates of the *i*-th reference point as $\left(x_{i},y_{i},0\right)$ (i.e., all the reference points are placed on a single plane, i.e., at z=0 in the proposed coordinate system) and the range estimate as $d_{i}$, the following set of equations holds true $\forall_{i}$ assuming that there is no range error.

(1) $$d_{i}^{2}={\left(x_{i}-x\right)}^{2}+{\left(y_{i}-y\right)}^{2}+z^{2}$$

For each reference point in the trilateration, a corresponding equation is used. Therefore, the position of the target can be found by solving these equations for the three unknowns (xy and *z*). Please note that although such setup (i.e., placing all the reference points on a single plane, i.e., at z=0 in the proposed coordinate system) will produce geometric dilution of precision (GDOP) [17], in our proposed method we used such kind of setup because (1) it requires minimum number of reference points (i.e., three reference points) to calculate 3D position of a target and (2) it is logistically simpler for indoor applications to place all the reference points on a single plane.

### 2.2. Cross-Correlation

The radii of the spheres required for the trilateration method are usually determined using the times taken for an US signal to travel from the transmitter to each receiver, referred to as the TOF of the signal, which are then translated into distances using the speed of sound. Cross-correlation is the standard digital signal processing technique for measuring the TOF. It is often simpler in practice to perform the calculation of the cross-correlation in frequency domain.

If the frequency domain transmitted and received signals are $S_{T}\left(f\right)$ and $S_{R}\left(f\right)$ respectively, the cross-correlation between them is given by:

(2) $$c\left(t\right)={ℑ}^{-1}\left(S_{T}^{*}\left(f\right)S_{R}\left(f\right)\right)$$

where ${ℑ}^{-1}$ is the inverse Fourier transform and * the complex conjugate. This cross-correlation provides a signal with a maximum value when the transmitted and received signals are perfectly aligned in time (which is the TOF) assuming no noise into the system.

Although cross-correlation in conjunction with a linear chirp signal provides superior performance [11], when multiple transmitters transmit chirp signals simultaneously MAI is introduced into the system which reduces system’s accuracy as discussed in the following section.

## 3. Description of Problem

If an UPS has *M* transmitters located at fixed known positions which simultaneously transmit a signal ($s_{T_{i}}\left(t\right)$, i=1,2,...,M), the signal received by a receiver in the system is [18]:

(3) $$s_{R}\left(t\right)=\sum_{i=1}^{M}A_{i}.\left(h_{i}*s_{T_{i}}\right)\left(t-t_{i}\right)+\tilde{n}\left(t\right)$$

where $A_{i}$ and $t_{i}$ are the respective amplitude and propagation delay (TOF) of the signal arriving from the *i*th transmitter and $\tilde{n}\left(t\right)$ the additive white Gaussian noise (AWGN), with the convolution operator (*) denoting the filtering effect produced by the US channel’s unknown impulse response ($h_{i}\left(t\right)$).

This ideal propagation model takes into account only the direct-path signal. However as, in an indoor environment, a receiver receives multiple delayed and attenuated replicas of a transmitted signal due to reflections from multiple objects in that environment, the impulse response of the *i*th transmitter can be modeled as:

(4) $$A_{i}\cdot \left(h_{i}*s_{T_{i}}\right)\left(t\right)≃\sum_{l=1}^{L_{i}}{\hat{A}}_{il}s_{T_{i}}\left(t-{\hat{t}}_{l}^{\prime }\right)$$

where $L_{i}$ represents the number of copies of the transmitted signal ($s_{T_{i}}\left(t\right)$) and ${\hat{A}}_{il}$ and ${\hat{t}}_{l}$ are the respective amplitude and propagation delay.

The received signal ($s_{R}\left(t\right)$) is processed using a matched filter implemented by correlating it with a reference signal ($s_{T_{k}}\left(t\right)$) (i.e., a locally stored copy of the original emitted signal) which results in:

(5) $$\begin{array}{c} c_{k}\left(t\right)=\left[s_{R}\left(t\right)★s_{T_{k}}\left(t\right)\right]\;\\ \;\;\;\;\;\;\;\;=\left(\sum_{i=1}^{M}A_{i}\cdot \left(h_{i}*s_{T_{i}}\right)\left(t-t_{i}\right)+\tilde{n}\left(t\right)\right)★s_{T_{k}}\left(t\right)\;\\ \;\;\;\;\;\;\;\;=(A_{k}\cdot \left(h_{k}*s_{T_{k}}\right)\left(t-t_{k}\right))★s_{T_{k}}\left(t\right)+\;\\ \;\;\;\;\;\;\;\;\;\left(\sum_{i\neq k}A_{i}\cdot \left(h_{i}*s_{T_{i}}\right)\left(t-t_{i}\right)\right)★s_{T_{k}}\left(t\right)+\tilde{n}\left(t\right)★s_{T_{k}}\left(t\right) \end{array}$$

In Equation (5), the first term on the right-hand side is the auto-correlation of the transmitted signal with itself which is distorted by the channel response ($h_{i}\left(t\right)$), and the second term represents the MAI from all the other transmitters simultaneously transmitting in the environment which are treated by $c_{k}\left(t\right)$ as noise because it follows a single-user approach.

Therefore, the earliest component of $c_{k}\left(t\right)$ is $\left[s_{k}★s_{k}\right]\left(t-t_{k}\right)$ (where ★ implies a correlation) the peak of which can be used to determine $t_{k}$ (the direct-path signal of $s_{k}\left(t\right)$) with considerable precision provided the other multipath components (from $s_{k}\left(t\right)$) of r(t) are sufficiently weak and/or separated in time from $t=t_{k}$. The MAI and noise (respectively, the second and third terms of Equation (5)) may shift the peak at $t_{k}$ from its actual timeline which could result in an inaccurate estimate of the range information.

Therefore, as MAI has a large effect on the accuracy of TOF estimations, it is often desirable that the cross-correlation between transmitted signals (the second term in Equation (5)) is as low as possible. In this paper, we present orthogonal chirp waveforms, in which multiple transmitters can simultaneously transmit chirp signals without any interference (i.e., the cross-correlation between transmitted signals is zero). Though the performance of the analogous waveform has been investigated through simulations in radar communications [19], still it has not been adapted for UPS.

## 4. Orthogonal Chirp Waveforms for Multiple Access Ultrasonic Positioning

The fundamental idea behind the proposed orthogonal chirp waveforms scheme for simultaneous multiple transducer positioning is to utilize the oversampling methodology of OFDM modulation and orthogonality of the discrete frequency components of a chirp waveform. In this section we present pictorial as well as mathematical representations of the orthogonal chirp waveforms scheme. A linear chirp is defined as:

(6) $$\begin{array}{cc} s\left(t\right)=\mathrm{rect}\left(\frac{t}{T_{c}}\right)\exp\left\{j2\pi \left(f_{s}t+\frac{1}{2}\mu t^{2}\right)+\phi \right\} & 0\leq t\leq T_{c} \end{array}$$

where rect (•) is a rectangular window function, $f_{s}$ the starting frequency, $T_{c}$ the chirp duration, ϕ the initial phase, $\mu =\frac{B}{T_{c}}$ the chirp rate (where bandwidth $B=f_{s}-f_{h}$ with $f_{s}$ and $f_{h}$ the starting and ending frequencies respectively of the chirp signal). The chirp signal in Equation (6) neglects the amplitude.

**Figure 1:**
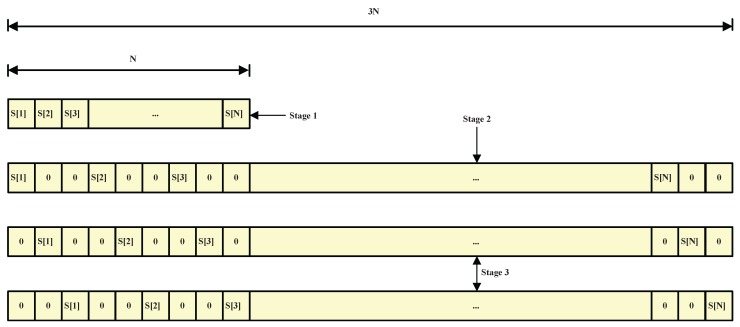
Illustrations of data sequence of discrete spectra allocated to transmitters.

Our proposed ultrasonic multiple access technique works in a three-stage process. In the first stage, the fast Fourier transform (FFT) of a chirp signal is placed in the discrete data sequence serially, as shown in Figure 1 where there are *N* discrete spectra (S[1],S[2],...,S[N]). Mathematically, the data sequence of a chirp spectrum (i.e., stage 1) is given by:

(7) S[p]=DFT[s[n]]

where s[n] represent the chirp signal in the discrete domain which can be obtained by introducing $t=nT_{s}$ in Equation (6) where n=1,2,...,N and $T_{s}$ is the sampling interval.

As the second stage involves interleaving M−1 zeros after each discrete spectrum, where *M* represents the number of transmitters used by an UPS, the new data sequence has MN discrete spectra, with the data sequence obtained dedicated to the first transmitter. Based on the example shown in Figure 1, if three transmitters are used by an UPS, we need to interleave two (M−1=3−1=2) zeros after each discrete spectrum. It is noted that, due to its zero interleaving , the length of the data sequence is increased from *N* to 3*N* (MN= 3*N*). In addition, it (zero interleaving) refers to the repetition of the signal (*M* times). Mathematically, in the time domain, the signal dedicated to the first transmitter (i.e., the time domain signal of stage 2) is defined as:

(8) $$\begin{array}{c} s_{1}\left(t\right)=s\left(t\right)\mathrm{rect}\left(\frac{t}{T_{c}}\right)+s\left(t-T_{c}\right)\mathrm{rect}\left(\frac{t-T_{c}}{T_{c}}\right)+\\ ...+s\left(t-\left(M-1\right)T_{c}\right)\mathrm{rect}\left(\frac{t-\left(M-1\right)T_{c}}{T_{c}}\right) \end{array}$$

In the third stage, the data sequence obtained in the second stage is shifted by *i* (i=1,...,M−1) to generate the remaining data sequences which are dedicated to the rest of the transmitters used by the UPS; for example, in Figure 1, the data sequence obtained from the second stage is initially shifted by one and then two to generate the other two data sequences. Mathematically, in the time domain, the signal dedicated to the remaining transmitters (i.e., the time domain signals of stage 3) is defined as:

(9) $$s_{i+1}\left(t\right)=s_{1}\left(t\right)e^{\left(j2\pi \frac{i}{MT_{c}}t\right)}$$

Although all these signals are transmitted in parallel from individual transmitters, the receivers receive them without any interference as the multiplication of their respective spectra results in zero which means they are orthogonal, as shown in Figure 1 for three chirp signals.

According to Equation (9), if *i* is increased, the number of orthogonal chirps will increase which will increase system capacity, however, at the same time, based on Equation (8), the length of each waveform will increase *M* times which will increase the processing time and system cost. In this paper as we generated three orthogonal chirp signals (i.e., M=3) from a 5 ms duration of a 35–45 kHz linear chirp s(t) (Equation (6)), the length of each orthogonal chirp waveform became 15 ms according to Equation (8). It is important to note that the design process is also valid for nonlinear chirp.

## 5. Performance Evaluation of Orthogonal Chirp Waveforms

**Figure 2:**
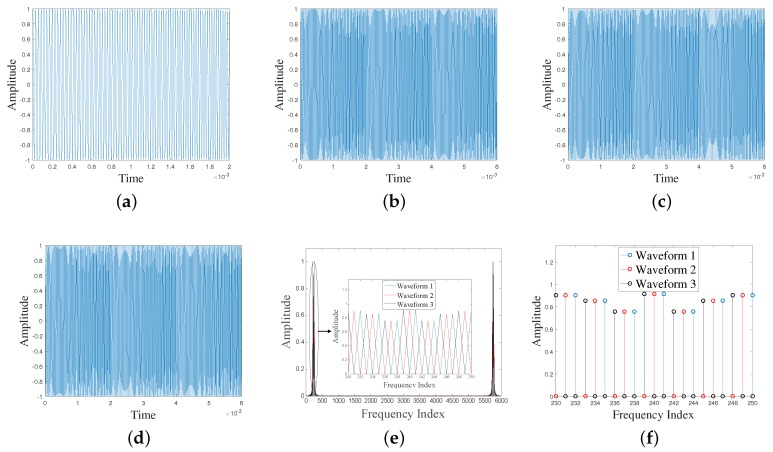
(**a**) 35–45 kHz/2 ms chirp in time domain with sampling frequency 1 MHz; (**b**–**d**) three orthogonal chirp waveforms respectively, generated from (**a**); (**e**) corresponding frequency spectra of the three orthogonal chirp waveforms shown in (**b**–**d**); and (**f**) stem graph of the zoom plot of (**e**).

Based on the chirp signal (generated using Equation (6) and shown in Figure 2a, examples of three (M=3) orthogonal chirps (generated using Equations (8) and (9)) are shown in Figure 2b–d. It is noted that, due to its zero padding, length of each waveform is increased by factors of 3 (i.e., *M*) from those of the original waveform (Figure 2a). It has been noticed that unlike OFDM signal, in the proposed scheme each waveform has constant modulus in time domain which will lead to low peak-to-average power ratio (PAPR) which is desire. Although, due to the narrowband nature of the 35–45 kHz/2 ms chirp, the repetition property of the chirp signal presented in the proposed scheme (described in earlier section) is not visible in time domain analysis (Figure 2b–d), it can be visualized in correlation analysis that is discussed later in this section. The spectra of the three orthogonal chirps are shown Figure 2e in which it is clear that each chirp is orthogonal to the others because the multiplication of their respective spectra results in zero. Moreover, we can see that the center frequency of each sub-carrier of each waveform occurs at a null in the spectra of all the other sub-carriers which means that they are as densely packed as possible like an OFDM system.

**Figure 3:**
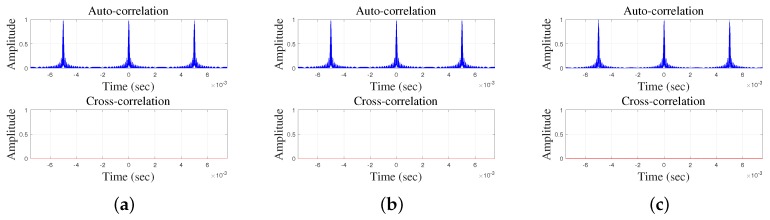
Comparisons of auto-correlations and cross-correlations of three waveforms for the proposed method: (**a**) waveforms 1 and 2; (**b**) waveforms 1 and 3; and (**c**) Waveforms 2 and 3.

As discussed in Section 3, for multiple access, an important property is the relative difference between the auto-correlations of identical waveforms and cross-correlations of different ones which now we investigate for the proposed orthogonal chirp waveforms scheme. For M=3, the relative differences between the auto-correlations of the identical waveforms and cross-correlations of different ones described in Section 4 are shown in Figure 3 where the orthogonal chirp waveforms were generated from a 35–45 kHz/5 ms chirp which also used in simulations and experiments. It can be seen that, due to the repetition property, the auto-correlations of all waveforms produce three main peaks while the cross-correlations are fully suppressed because, as the spectral components of the three waveforms are mutually shifted by $\frac{1}{MT_{c}}=\frac{1}{3T_{c}}$, they are fully orthogonal to each other.

As, according to Figure 3, the cross-correlations between transmitted signals (the second term in Equation (5)) are fully suppressed (i.e., zero), the MAI (second term in Equation (5)) will have no effect. Therefore, one could accurately calculate the TOF even though multiple transmitters transmit signals simultaneously.

However, it is important to note that as there are multiple peaks involve in the correlation process, the peak generated by the reflected path may exceed the main one of $c_{k}\left(t\right)$ (i.e., cross-correlation in Equation (5)) due to multipath, MAI and noise, and that associated with the correct delay is not always the highest one. In some cases, the direct path can experience attenuation, which gives it a lower cross-correlation peak than indirect multi-paths. In other cases, a number of indirect paths can combine to produce a peak that is greater than the one associated with the direct path. Therefore, a threshold-based search mechanism [20] is applied to find the first cross-correlation peak to arrive above the noise floor which is assumed to belong to the direct path that gives the correct TOF. The threshold is set to 70% of the height of the cross-correlation peak as that value is found to be sufficiently high (through experiments) to detect this early peak and sufficiently low to guarantee detection of the direct-path peak, even with strong reflections.

## 6. Simulation Results

A customized environment was simulated in Matlab to evaluate the performance of the proposed waveform schemes for multiple access in an UPS. In a virtual 3D rectangular room, in a passive mobile architecture, three reference points (with known locations) were considered and a target introduced as a receiver with the aim of localizing it, with the true positions of the receiver known. Table 1 shows the coordinates (x,y,z) of the room, reference points, and target.

According to the procedure described in Section 4, with sampling rate of 1 Msample/s, three orthogonal chirp waveforms, were generated from a 35–45 kHz/5 ms chirp signal. These orthogonal chirps were also used for the actual experiments. The three waveforms were transmitted simultaneously from the reference points (each of which was modelled with a bandpass (35–45 kHz) filter) and received by the receiver, the position of which was calculated using the trilateration algorithm described in Section 2.1. The distance information used in this algorithm was obtained using the TOF ($t_{k}$) information, calculated according to the frequency–domain cross–correlation described in Section 2.2 along with the threshold-based earliest correlation peak search mechanism described in Section 5. It is important to note that, although we considered one target in this simulation, it would be possible to localize arbitrary number of targets using this approach as, in a passive mobile architecture, the wireless channel is not dependent on the number of targets. For the simulation, it was assumed that the channel was subjected to additive white Gaussian noise (AWGN) with SNRs of 0 dB and six multi-paths at random positions with reflection coefficients of 0.7, and each was run for 20,000 iterations. Please note that although we considered multipaths at random position, it was ensured that the minimum separation between each path was larger than 1/B sec because chirp signals sweeping *B* Hz can resolve two different chirp signals traversing with 1/B sec path difference [21].

**Figure 4:**
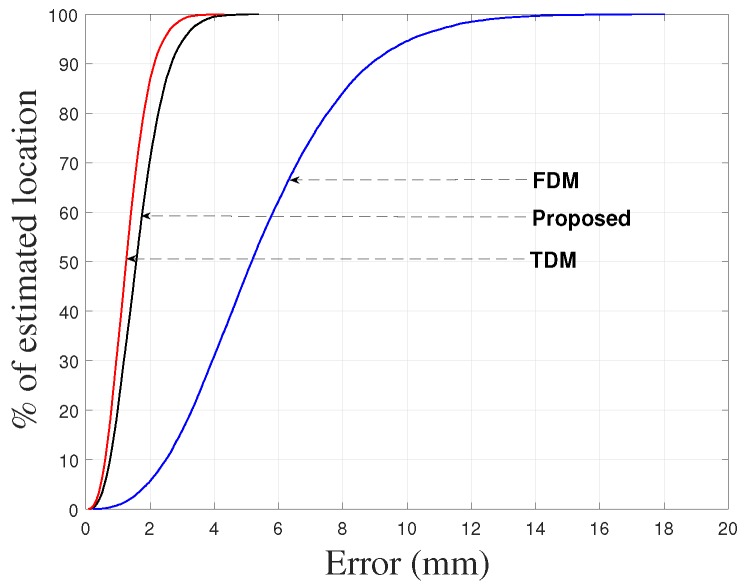
Cumulative absolute location errors (from simulations) of target (receiver) obtained for the proposed, TDM and FDM techniques.

To demonstrate the performance of the proposed approach, the positions of the receiver were also calculated by the TDM and FDM techniques using 35–45 kHz/5 ms chirp. For the TDM technique the orthogonality was maintained by transmitting the same pulse from collocated transmitters at different times whereas for the FDM technique the orthogonality was maintained by splitting the bandwidth to the transmitters equally. The cumulative absolute location errors of the receiver for the proposed method, TDM, and FDM techniques are shown in Figure 4, with the 90% error presented in Table 2 (for the proposed, TDM and FDM techniques) in which the RMSEs of the absolute location errors of the receiver are also given. The deviation in accuracy for TDM and FDM techniques is noticeable due to the reasons described in Section 1. The results indicate that the proposed system is comparable with the TDM technique.

## 7. Experimental Procedure

To evaluate the performance of the proposed waveform scheme for multiple access in an UPS, experiments were conducted in indoor noisy and multi-path environments. In a passive mobile architecture three reference points (transmitters) with known locations were considered and nine targets (receivers) were introduced with the aim of localizing them. Please note that all the receivers were placed on a single plane (approximately 1000 mm away from the reference points) and the the gap between them was 5 cm (with a precision of 0.3 mm).

**Figure 5:**
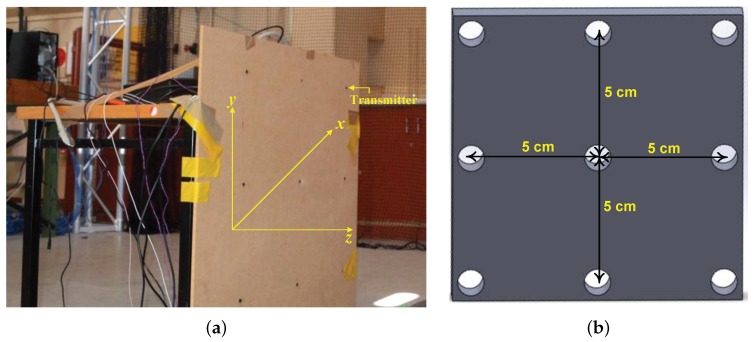
Experimental setup: (**a**) configuration of reference plane; and (**b**) targets.

The configurations of the reference points and targets are shown in Figure 5a,b respectively. In Figure 5a although 9 transmitters (reference points) are visible, only the side ones in the middle row and central one in the top row were used. Piezotite MA40S4S and MA40S4R US devices, which centre frequency is around at 40 kHz, were used as transmitters and receivers respectively. According to the procedure described in Section 4, three orthogonal chirp waveforms were generated. As for the simulation, all the waveforms were generated from a 35–45 kHz/5 ms chirp signal.

**Figure 6:**
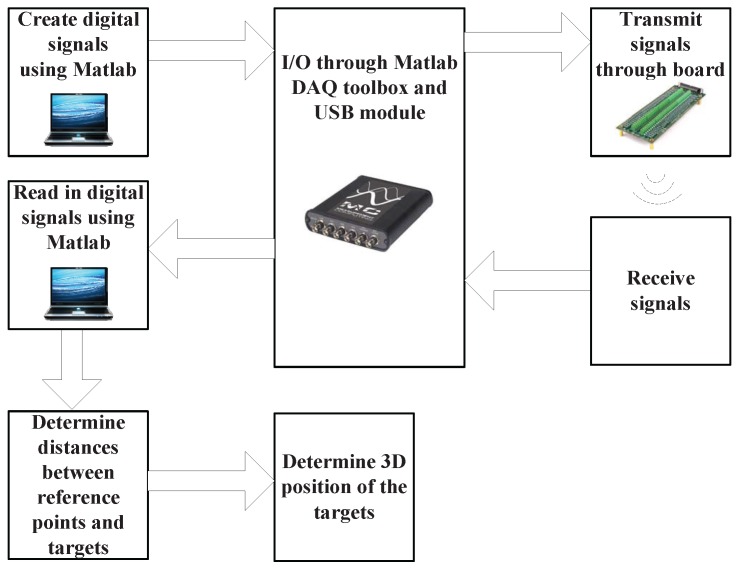
Illustration of the experimental procedure.

The three waveforms were distributed to the reference points and transmitted simultaneously with the aim of localizing the receivers. The experimental process is shown in Figure 6. The input signals into the transmitters were from a screw pin board [22] which was connected to a Measurement Computing USB-1604 data acquisition (DAQ) module (the sampling rate of which was 1 Msample/s) [22]. The DAQ was also connected to a laptop and hence Matlab, in order to be able to send the transmission signals. This configuration also allowed for the capture of the received signal using the DAQ and the DAQ tool boxes in Matlab. The setup was the same for every set of orthogonal chirp waveforms and each location measurement was repeated 100 times. Please note that for a positioning system which requires a very high degree of accuracy (millimeter), the selection of its data acquisition (DAQ) module was an important issue. In order to determine an appropriate interface for the positioning system, potential detection errors were studied under a specific scenario which involves investigating how many errors will be introduced if the time detection is delayed by one sample which helps to ascertain the sampling rate to be used to maintain millimeter accuracy. Using the common audio interface, the sampling rate of which is 100 kHz, i.e., a sampling time of $T_{s}=\frac{1}{100\;\mathrm{kHz}}=10\;\mu s$, if the detection is out by one sample period, when the speed of sound is 344m/s, the prospective error in the predicted distance ($d_{e}$) will be:

(10) $$d_{e}=v\times T_{s}=10\;\mu s\times 344\;m/s\approx 3.6\;\mathrm{mm}$$

Although this error is too large for a highly accurate UPS, it can be either physically or virtually reduced by increasing the sampling rate. As, if the sampling rate is increased virtually, e.g., through interpolation, the complexity and uncertainty in the system’s software is increased, the choice was to increase it physically. As the desired accuracy is less than 0.5 mm, according to Equation (10), the sampling rate has to be $T_{s}=\frac{0.5\;\mathrm{mm}}{344\;m/s}=1.45\;\mu s$ which corresponds to a minimum sampling frequency of $f_{a_{\min}}=\frac{1}{1.45\;\mu s}\approx \;0.7\;\mathrm{MHz}$.

Therefore, in order to obtain accurate data simultaneously at each receiver, the minimum sampling frequency must be 0.7 MHz. After conducting market research, the USB-1604HS-2AO DAQ module [22] was selected as it has simultaneous 1.33 MHz sampling at each of its four input channels and 1 MHz sampling at its output channel which means that the error ($d_{e}$) incurred by one sample’s false detection is reduced to 0.344 mm, a huge improvement compared with typical audio interfaces.

To demonstrate the performance of the proposed approach, the positions of the targets (receivers) were also calculated by the TDM and FDM techniques using 35–45 kHz/5 ms linear chirp. As the sound velocity depends on temperature, to measure the room temperature a digital thermometer was used and the corresponding velocity was measured. The measured temperature (φ) was 23 °C and its corresponding velocity (v) was calculated as 345.10 m/s using the formula v=(331.3+0.6φ)m/s. Since the effect of humidity on the speed of sound is much smaller than for temperature, the effect of humidity on sound velocity was assumed to be negligible. Please note that as the measurements were taken over a short period of time, the effects of variations in temperature and humidity on the sound’s velocity were assumed to be negligible. Therefore, the sound velocity was assumed to be constant during the experiments.

## 8. Results and Discussions

**Figure 7:**
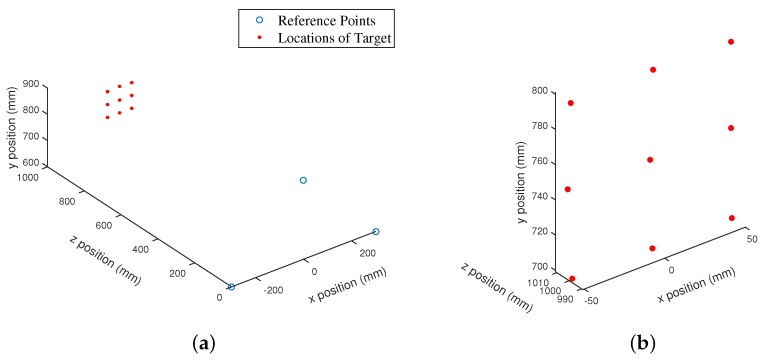
(**a**) A test case from the experimental results that represents the positioning of transmitters and receivers; and (**b**) zoomed version of the position of the receivers.

**Figure 8:**
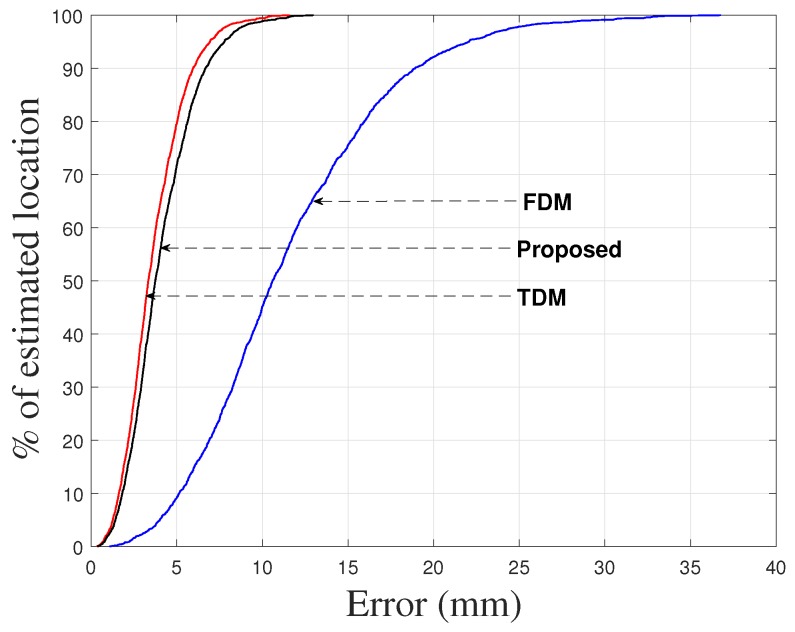
Cumulative absolute location errors (from experiments) of receiver obtained for the proposed, TDM and FDM techniques.

**Figure 9:**
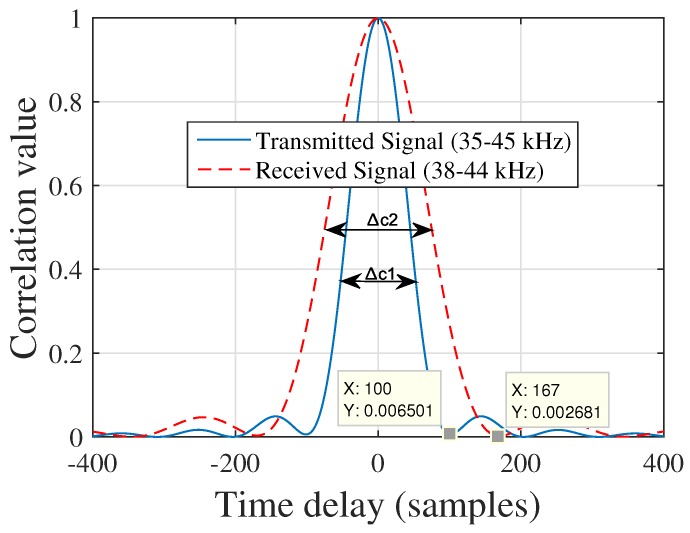
Auto-correlation width of the transmitted and received signal where the correlation width (Δc1) 100 and (Δc2) 167 refers 10 kHz and 6 kHz respectively according to the formula $\Delta c≃\pm \frac{1}{T_{s}B}$ [15].

After completing the experiments for a location of the receiver plane in a passive mobile architectures for multiple access chirp-based ultrasonic positioning, the position of each target was calculated using the same procedure discussed in Section 6. A test case from the experimental measurements for the location of the receiver plane is shown in Figure 7. The absolute location errors of the receiver obtained from the experiments for the proposed, TDM, and FDM techniques are shown in Figure 8 in terms of 90% error. The 90% error for the proposed approach, TDM and FDM techniques is summarized in Table 2 along with RMSEs. The positioning accuracies of the TDM and FDM techniques were higher and lower for the reason described in Section 1 and the accuracy of the proposed method is comparable with the TDM technique. It has been noticed that the experimental results (Figure 8, Table 2) have been degraded when compare to simulation results (Figure 4, Table 2). This is because the bandwidth restriction imposed by resonant transducers i.e., the transducers had not enough bandwidth to transmit the assigned chirp signals. As it is well known that for a linear chirp the correlation width (Δc) is inversely proportional to the bandwidth (*B*) (i.e., $\Delta c\propto \pm \frac{1}{B}$), for the experimental data (for TDM technique) we compared the auto-correlation width of a transmitted and received linear chirp signal in Figure 9 where we can see that although we transmitted a signal with 10 kHz bandwidth, we received the signal with a 6 kHz bandwidth (centre frequency was 40 kHz) which indicates the bandwidth restriction imposed by resonant transducers.

In our current setup we did not consider moving objects. However, it is possible to localize moving objects using the proposed orthogonal chirp waveforms. For example, in our current setup, according to the procedure described in Section 4, three orthogonal chirp waveforms were generated from a 35–45 kHz/5 ms chirp. Thus, the length of each orthogonal chirp waveform was increased to 15 ms which represents a frequency resolution of approximately $f_{b}=1/T_{s}=66.67$ Hz. Therefore, for the current setup if we wished to produce this frequency shift into 40 kHz ($f_{c}$), for speed of sound (*v*) is 345.1 m/s (which was measured during experiments), the maximum target’s velocity ($v_{t}$) with Doppler tolerance would be $v_{t}=\frac{f_{b}\times v}{f_{c}}=\frac{66.67\;\mathrm{Hz}\times 345.1\;m/s}{40000\;\mathrm{Hz}}=0.58$ m/s. Although this Doppler tolerance is low for larger-scale indoor positioning applications, e.g., human navigation and robotic navigation, one can improve the Doppler tolerance by increasing the sampling frequency, in other word, the sampling rate $T_{s}$ which can be achieved either virtually or physically. Advanced signal processing technique such as [19] can also be employed to improve the Doppler tolerance which does not require to increase the sampling frequency.

## 9. Conclusions and Future Work

In this paper, to facilitate multiple access transmission in a chirp-based UPS, we proposed to use orthogonal chirp waveforms, in which multiple transmitters can simultaneously transmit chirp signals, as a result, it can efficiently utilize the entire available frequency spectrum. In addition, the proposed orthogonal chirp waveforms have all the advantages of classical chirp waveform. As, for simultaneous transmission, the proposed approach did not use either a TDM or FDM technique, there were no impacts on the system’s update rate or cross-correlation performance (in terms of accuracy).

The performance of the proposed method was experimentally validated for a passive mobile architecture over an operational range of approximately 1000 mm, with their positioning RMSEs and cumulative errors (for 90% of cases) were 4.54 mm and 6.68 mm respectively.

It is worth noting that, although the proposed method achieved greater accuracy in an indoor environment for static target localization, for moving object localization its performance has not been investigated yet. Therefore, our future work involves incorporating advanced signal processing such as [19] so that the proposed system can be used for larger-scale indoor positioning applications, e.g., human navigation, with the goal of directing users to their desired destinations on an active map, and robotic navigation, where location sensors provide position information to a moving robot. 

## Figures and Tables

**Table 1:** Coordinates of the 3D rectangular room, and ideal positions of the reference points and target transducer (cm).

	*x*	*y*	*z*
Room (top left corner)	−300	300	0
Room (bottom right corner)	300	0	0
Reference Point 1	−60	60	0
Reference Point 2	60	60	0
Reference Point 3	0	120	0
Target	−5	95	100

**Table 2:** Absolute location errors (from simulations and experiments) of receiver obtained for the proposed, TDM and FDM techniques in terms of 90% Error and RMSE (mm).

Technique	Proposed	TDM	FDM
RMSE & 90% Error (from simulations)	1.82 & 2.67	1.45 & 2.13	6.04 & 8.87
RMSE & 90% Error (from experiments)	4.54 & 6.68	4.05 & 5.95	12.86 & 18.92

## Abbreviations

The following abbreviations are used in this manuscript:

3D	three-dimensional
CDMA	code division multiple access
DSSS	direct sequence spread spectrum
FDM	frequency-division multiplixing
FHSS	frequency hopped spread spectrum
FM	frequency-modulated
MAI	multiple-access interference
OFDM	orthogonal frequency-division multiplixing
RMSE	root-mean-square-error
SNR	signal-to-noise ratio
TDM	time-division multiplixing
TOF	time-of-flight
UPS	ultrasonic positioning system
US	ultrasonic
